# Effects of Kynurenic Acid on the Rat Aorta Ischemia—Reperfusion Model: Pharmacological Characterization and Proteomic Profiling

**DOI:** 10.3390/molecules26102845

**Published:** 2021-05-11

**Authors:** Viviane Soares Souza Lima, Douglas Oscar Ceolin Mariano, Hugo Vigerelli, Sabrina Cardoso Janussi, Thayz Vanalli Lima Baptista, Mário Angelo Claudino, Daniel Carvalho Pimenta, Juliana Mozer Sciani

**Affiliations:** 1 Laboratório Multidisciplinar em Pesquisa, Universidade São Francisco, 12916-900 Bragança Paulista, Brazil; viviane.soares@usf.edu.br (V.S.S.L.); s.aa.h@hotmail.com (S.C.J.); thayzvanali@hotmail.com (T.V.L.B.); mario.claudino@usf.edu.br (M.A.C.); 2 Laboratório de Bioquímica e Biofísica, Instituto Butantan, 05503-900 São Paulo, Brazil; douglasceolin@gmail.com (D.O.C.M.); dcpimenta@butantan.gov.br (D.C.P.); 3 Laboratório de Genética, Instituto Butantan, 05503-900 São Paulo, Brazil; hugo.barros@butantan.gov.br

**Keywords:** kynurenic acid, ischemia-reperfusion, aorta, functionality, proteomics

## Abstract

Kynurenic acid (KYNA) is derived from tryptophan, formed by the kynurenic pathway. KYNA is being widely studied as a biomarker for neurological and cardiovascular diseases, as it is found in ischemic conditions as a protective agent; however, little is known about its effect after ischemia-reperfusion in the vascular system. We induced ischemia for 30 min followed by 5 min reperfusion (I/R) in the rat aorta for KYNA evaluation using functional assays combined with proteomics. KYNA recovered the exacerbated contraction induced by phenylephrine and relaxation induced by acetylcholine or sodium nitroprussiate in the I/R aorta, with vessel responses returning to values observed without I/R. The functional recovery can be related to the antioxidant activity of KYNA, which may be acting on the endothelium-injury prevention, especially during reperfusion, and to proteins that regulate neurotransmission and cell repair/growth, expressed after the KYNA treatment. These proteins interacted in a network, confirming a protein profile expression for endothelium and neuron repair after I/R. Thus, the KYNA treatment had the ability to recover the functionality of injured ischemic-reperfusion aorta, by tissue repairing and control of neurotransmitter release, which reinforces its role in the post-ischemic condition, and can be useful in the treatment of such disease.

## 1. Introduction

Kynurenic acid (KYNA) is one of the metabolites produced along the kynurenine pathway, an event that occurs in tryptophan catabolism to form NAD or NADH. Basically, the reaction steps involve the formation of formylkynurenine by the action of either tryptophan 2,3-dioxygenase or indoleamine 2,3-dioxygenase, followed by the hydrolysis of formylkynurenine to kynurenine catalyzed by formamidase and its transamination into KYNA by kynurenine amino transferases [1,2]. The availability of kynurenine is the limiting factor for the KYNA formation reaction, as the amino transferases have low affinity for the substrate [3].

Synthesis of KYNA by heart, kidney, and liver was already reported [4] and in vivo studies showed the presence of KYNA in serum, being probably synthesized by endothelium after hypoxia events [5].

KYNA acts in several targets: it is agonist of G-protein-coupled receptor 35 and aryl hydrocarbon receptor; antagonist of AMPA/kainite receptors, glycine co-agonist site of NMDA receptors (at low concentrations) and α-7 nicotinic receptors. Moreover, it has scavenger of hydroxyl radical, peroxynitrite and superoxide [6,7].

Due to its binding on AMPA and α-7 nicotinic receptors, critically involved in neurological processes, such as neurodevelopment, plasticity, cognition, behavior, and memory, the effects of KYNA on the central nervous system (CNS) is being explored since the late 70s, as well as its participation in neurological diseases [8].

The concentration of KYNA in the human CNS has been estimated in a range of 15 to 150 nM, values that can oscillate according to behavioral and cognitive alterations—low concentration of KYNA in the brain cause improvement of cognitive processes in animal models. [7,9]. On the other hand, the reduction of KYNA concentration in the brain can result in the in-crease of excitability mediated by glutamate, causing neurological diseases. Due to these effects, KYNA has been widely studied as a neuroprotector agent [10].

Besides activities on the CNS, KYNA effects are being reported in the periphery by binding in the G-protein coupled receptor (GPR35) and aryl hydrocarbon receptor (AHR). GPR35 is distributed in lung, stomach, small intestine, colon, spleen, and cells of the immune system, such as macrophages and monocytes. In immune cells, when activated, reduces pro-inflammatory cytokines release [11]. The activation of AHR also influences the immune system in inflammatory, infectious, and neoplastic disorders in certain tissues and cells, such as colon, kidney, and dendritic cells. The activation and differentiation of T-cells by IL-6 and IL-21 is one pro-posed mechanism for this receptor modulation [12,13].

Metabolites produced in the kynurenine pathway, including KYNA, can also exert important roles in cardiovascular physiology and pathology [14]. Kynurenine, anthranilic acid, 3-hydroxykynurenine, and xanthurenic acid were found increased in patients after cardiovascular disease mortality [15]. Pedersen et al. [16] showed that the increase of kynurenines concentration in plasma can predict an increased risk of acute myocardial infarction in patients with suspected stable angina pectoris. Remote ischemic conditioning (RIC) in rats induced KYNA synthesis, in a mechanism dependent on tryptophan-kynurenine pathway activation in the liver, which exerted cardio-protective effects [17].

Due to this correlation between KYNA and pathologies, the metabolite is being considered a biomarker for cardiovascular diseases [18]. Furthermore, KYNA levels in blood can be used to predict death and recurrent myocardial infarction in patients with coronary artery disease [19].

Besides pathologies prediction, studies demonstrated that KYNA can act in tissue repair. Nahomi et al. showed that higher levels of KYNA protected retinal ganglion cells from ischemia-reperfusion (I/R)-induced damage [20]. Moreover, preclinical studies suggested that treatment with kynurenine and kynurenine-3-monooxygenase inhibitors de-creased infarct size [21].

In addition, KYNA could prevent hypotension, when administrated intravenously before the induction of heatstroke in rats [22]. This fact raised the point that the molecule could be useful as a treatment for several cardiovascular diseases.

Cardiovascular diseases are one of the majority causes of death or morbidity. The economic costs to prevent or treat heart diseases are substantial and constitute a major concern for public health policy [23].

Ischemic disease is the most prevalent among cardiovascular diseases and it is characterized by the narrow (vasoconstriction) of arteries, or even total occlusion, decreasing or interrupting the blood supply for the tissues. In the heart, the prolonged lack of nutrients and oxygen can result in myocardial infarction, defined as cell death by necrosis of the cardiac muscle [24]

The aorta ischemia-reperfusion (I/R) can be found in several clinical conditions, such as abdominal aorta aneurism, acute thromboembolism followed by aortic atherosclerosis, and trauma surgery [25].

The lack of oxygenation for the tissues shifts the metabolism from aerobic to anaerobic, with high consumption of high energy substrates (ATP). When the blood flow is reestablished, a phenomenon known as reperfusion, the production of reactive oxygen species, inflammatory mediators, and alterations in the cell metabolism occurs, leading to the generation of toxic compounds that exacerbate the injury of the tissue [25,26].

The consequences for the affected tissue depend on the event duration, the extent of the ischemic injury, and the efficiency of the reperfusion [26,27]. Nevertheless, the tissue that received the blood supply is not the only organ affected, but also the blood vessel. Studies showed that vasomotricity and endothelial functions are affected by ischemia-reperfusion (I/R), impairing the patient’s prognosis [28].

Taking this into account, the understanding of molecular alterations during I/R is essential, as well as alternatives for its treatment. In this work we evaluated the effects of KYNA in the rat aorta submitted to I/R, to check its functionality through pharmacological tools, combined to proteomics approaches to clarify the mechanisms of action of the molecule, never studied before.

## 2. Results

### 2.1. I/R Model

**Figure 1:**
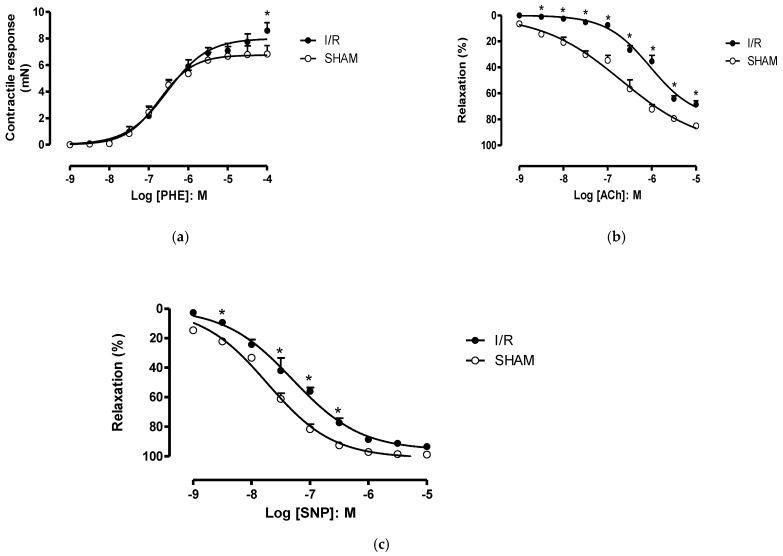
Rat aorta response after I/R. (**a**) Contractile response induced by phenylephrine (PHE). (**b**) Relaxation response induced by acetylcholine (ACh). (**c**) Relaxation response induced by sodium nitroprussiate (SNP). * *p* < 0.05.

To evaluate the contractile mechanism, the cumulative addition of the alpha-1 adrenergic receptor agonist phenylephrine (PHE) caused a concentration-dependent contraction of the rat aorta, in all groups. The ischemia-reperfusion (I/R) significative increased the maximal response induced by PHE, when compared to sham (Figure 1A). The aorta ring relaxation was altered as well in the I/R group: both agonists acetylcholine, for non-selective muscarinic receptor (−8.5 to −5.0 M) and sodium nitroprussiate (−8.5 to −6.5 M), a nitric oxide donor, caused lower relaxation in the ischemic vessel (Figure 1B,C, respectively).

### 2.2. Effect of KYNA on Ischemic Aorta Contraction and Relaxation

**Figure 2:**
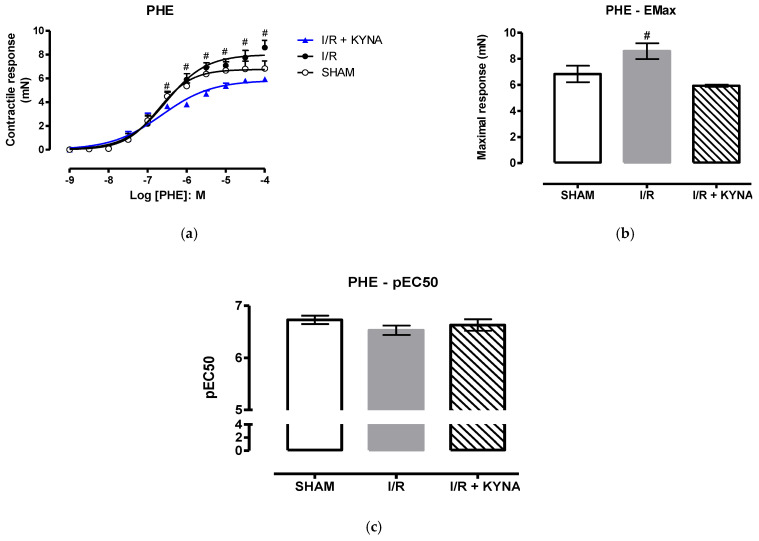
Rat aorta contraction induced by phenylephrine (PHE) after I/R and 100 µM KYNA treatment. (**a**) Concentration-response curve of PHE. (**b**) Maximum effect (Emax) of PHE. (**c**) Half maximal effective concentration (pEC50) of PHE. # *p* < 0,05 for I/R and I/R + KYNA comparison.

After KYNA incubation with ischemic aorta, less exacerbated contraction induced by cumulative addition of the agonist phenylephrine was observed, compared to the ischemic and not treated group (I/R), being statistically significant in high concentrations, as depicted in the concentration-response curve of aorta contraction (Figure 2A). The contractile response of aorta treated with KYNA was similar to the Sham group, aorta with no treatment and ischemia.

The Emax of I/R was statistically higher than KYNA (Figure 2B) and sham (Figure 2C) groups, but the pEC50 was not altered with the treatment.

**Figure 3:**
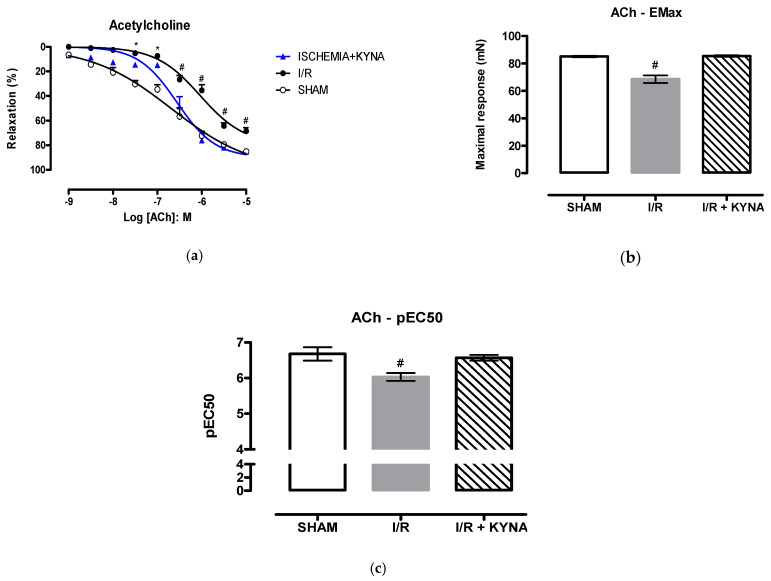
Rat aorta relaxation induced by acetylcholine (ACh) after I/R and 100 µM KYNA treatment. (**a**) Concentration-response curve of ACh. (**b**) Maximum effect (E_max_) of ACh. (**c**) Half maximal effective concentration (pEC_50_) of ACh. * *p* < 0.05 for Sham and I/R + KYNA comparison. # *p* < 0.05 for I/R and I/R + KYNA comparison.

To evaluate the relaxation, acetylcholine (Ach) was applied, and according to the concentration-response curve shown in Figure 3A, a high concentration of the agonist caused similar relaxation in the sham and I/R+KYNA groups, both different from I/R, which presented less % of aorta relaxation. Both E_max_ and pEC_50_ were significantly lower in the I/R group (Figure 3B,C, respectively).

**Figure 4:**
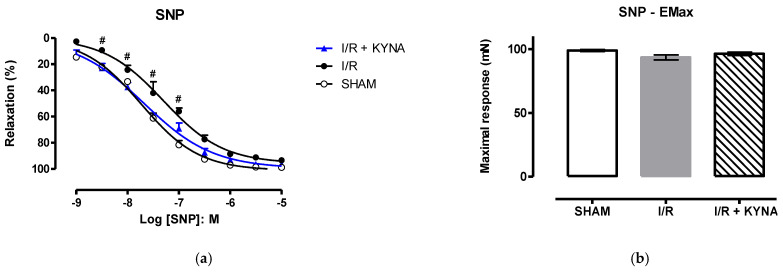

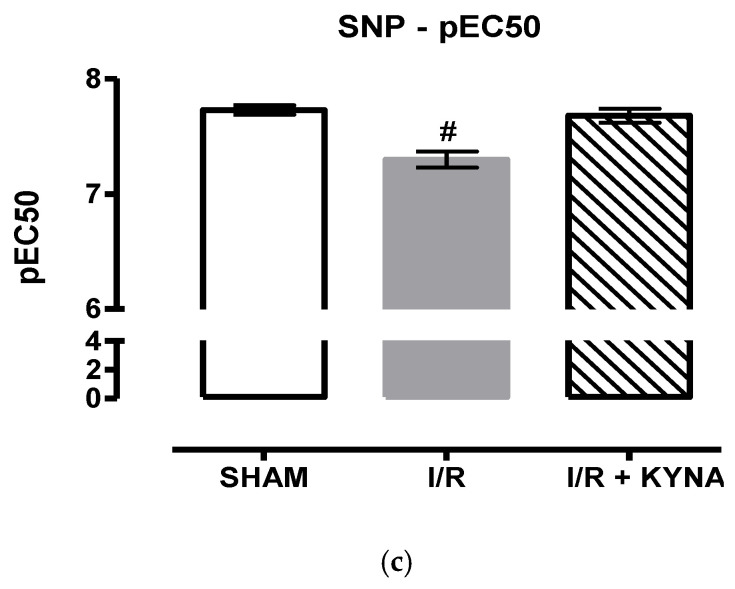
Rat aorta relaxation induced by sodium nitroprussiate (SNP) after I/R and 100 µM KYNA treatment. (**a**) Concentration-response curve of SNP. (**b**) Maximum effect (E_max_) of SNP. (**c**) Half maximal effective concentration (pEC_50_) of SNP. * *p* < 0.05 for Sham and I/R + KYNA comparison. # *p* < 0.05 for I/R and I/R + KYNA comparison.

When sodium nitroprussiate (SNP) was used, a similar profile was observed: less relaxation % in the I/R group compared to sham and I/R+KYNA groups. However, the difference was significant in lower concentrations (Figure 4A). In this case, only pEC50 was altered in the I/R group (Figure 4C), while the Emax was the same in all groups (Figure 4B).

### 2.3. Protein Profile f Ischemic Aorta Treated with KYNA

Through proteomic analysis, it was possible to identify 42 proteins in the I/R group and 135 proteins in the ischemic aorta treated with KYNA. In the I/R group, structural proteins were abundant, such as actin, tropomyosin, myosin, microtubules, and collagen. In KYNA-treated group, these structural proteins were found as well, besides proteins related to metabolism and regulation (Supplementary Material—Table S1).

**Figure 5:**
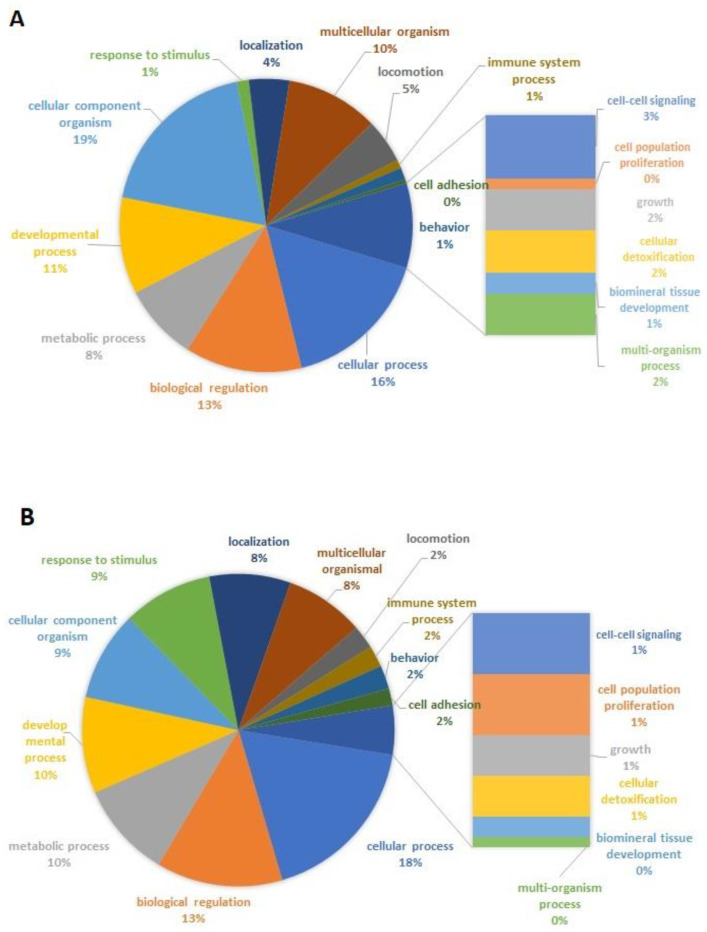
Biological process classification of proteins expressed in rat aorta after ischemia and reperfusion (I/R), treated or not with kynurenic acid. (**A**) I/R group. (**B**) I/R and treatment with 100 µM KYNA.

In order to understand the contribution of KYNA for the cell/tissue repair, the identified proteins were classified according to their biological process. Figure 5 shows their distribution into this classification and the comparison between I/R group (Figure 5A) and I/R+KYNA (Figure 5B). It is possible to observe that the KYNA treatment could change the protein expression profile, causing an increase of cellular processes, metabolic and immune cell processes, response to stimulus, localization, and cell adhesion and differentiation. Cellular component, cell-cell signaling, growth, biomineral tissue development, multicellular organismal and locomotion proteins were reduced with the treatment.

**Figure 6:**
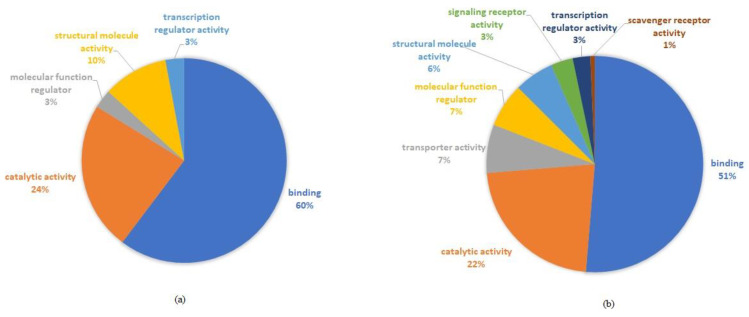
Molecular function of proteins from rat aorta after ischemia and reperfusion (I/R) and KYNA treatment. (**a**) I/R group. (**b**) I/R and treatment with 100 µM KYNA.

Regarding the molecular function, the protein profile was also altered after KYNA treatment, as shown in Figure 6.

Proteins related to binding activities were decreased with KYNA treatment (Figure 6B). Besides that, proteins related to catalytic and structural molecule activities were decreased when compared with control group (aorta artery submitted only to the I/R procedure) (Figure 6A). On the other hand, we observed an increase in proteins related to molecular function regulator, scavenging and signaling receptor activity, and molecular function regulator with KYNA treatment.

It is important to mention that proteins related to neurotransmitter release, synapsis, and neurogenesis were also found, as well as proteins that control calcium and potassium levels after I/R and KYNA treatment.

### 2.4. Protein-Protein Interaction

**Figure 7:**
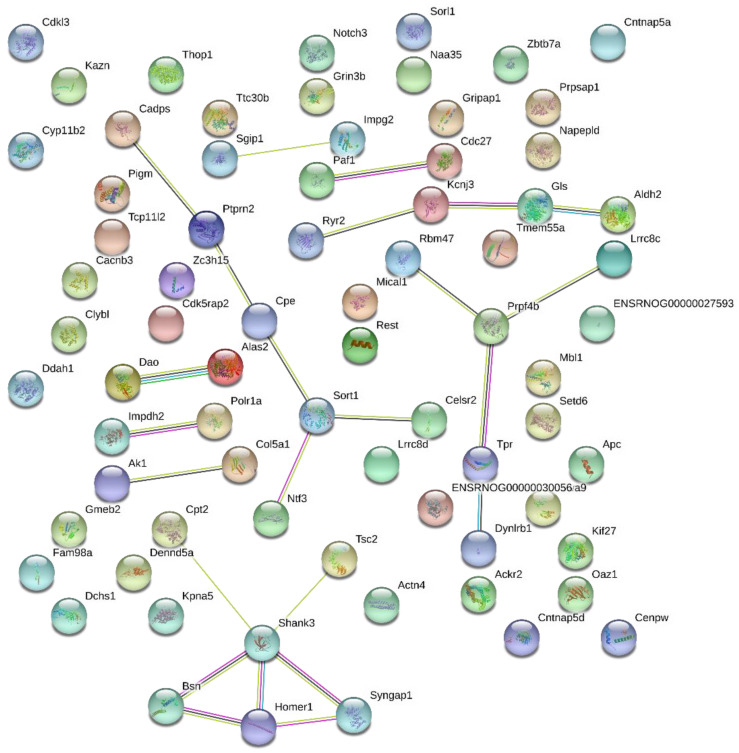
Protein-protein interaction networks of proteins from ischemic aorta treated with 100 µM KYNA. In this network, proteins are represented by nodes and lines represent functional associations between them (blue from curated database, pink experimentally determined, yellow is text mining, green gene neighborhood and dark blue gee co-occurrence).

Proteins from ischemic aorta treated with KYNA were analyzed by the STRING grid analysis, in order to find a functional link between them, as shown in the nodes from Figure 7. It was found that 73 proteins could be aligned to the STRING database, restricted to *Rattus norvegicus*.

Protein-protein interactions could be identified according to the edges depicted in Figure 7. The black edge represents a network of co-expressed proteins related to neurotransmitter release, neuron recovery and growth, and neurogenesis (Cadps, Ptprn2, Cpe, Sort1, Ntf3 and Celsr2). Another important network identified was the one highlighted with pink edges, in which proteins were experimentally determined and are related to synapse formation, molecules transportation through microtubules, calcium and neurotransmitter release, and post synapse signaling (Cpt2, Shank3, Tsc2, Bsn, Homer1 and Syngap1).

## 3. Discussion

Kynurenic acid is known for its activity in the central nervous system, modulating glutamatergic and cholinergic neurotransmission [29]. In addition, this molecule is being studied as a biomarker for cardiovascular diseases involving blood supply interruption, as it is believed that KYNA is produced for cell protection after an injury caused by ischemia and reperfusion [30].

However, its presence in the periphery is poorly studied, being reported in the heart, kidneys, liver, and endothelium [4,5,31]. Hypoglycemia and anoxia are factors that control the KYNA levels in serum, when produced by the endothelium [5,32].

Burkley, [33], postulated in 1987 that the ischemia itself, and consequently oxygen loss, was not the only factor responsible for the vascular damage, but the participation of toxic metabolites was essential for the injury.

The main toxic metabolites produced during I/R are the reactive oxygen species (ROS), such as hydrogen peroxidase, superoxide radical (^∙^O−2) and hydroxyl radical, generated from xanthine oxidase. The endothelium is susceptible to damage caused by ROS and its disfunction is characterized by the reduction of endothelium-derived relaxing factor (EDFR) levels [34]. Besides ROS production, pro-inflammatory mediators are released as well, contributing to the blood vessel lesion, especially in the lungs, kidneys, and heart [35].

Gross et al. [36] showed that ROS impaired the relaxation endothelium- dependent, and this activity could be restored by antioxidant enzymes like superoxide dismutase and catalase. KYNA has antioxidant proprieties and can prevent ROS formation by acting on antioxidant enzymes, as superoxide dismutase and catalase, besides reducing the pro-inflammatory cytokines IL-1β and IL-6 [37]. Moreover, Lugo-Huitrón et al. [38] showed that 100 to 300 μM KYNA had electron scavenger activity, able to inhibit oxidative stress.

Moroni et al., also demonstrated the reduction of proinflammatory cytokines release caused by KYNA, such as TNFa and HMGB1 from macrophages, in events like sepsis [39].

Inflammation is frequently observed in the reperfusion after an ischemic heart dis-ease for tissue remodeling, by the release of cytokines or due to the expression of ASC gene after NLRP3 inflammasome activation [40]. Therefore, by controlling the inflammation, the injured tissue can be remodeling. Fusco et al. showed that N-palmitoyl- ethanolamide-oxazoline (PEA-OXA), an anti-inflammatory and potent neuroprotective molecule, was able to reduce the injured area of the brain after ischemia, in an animal model, by decreasing the expression of NF-κB, cytokines, and neurotrophic factors [41].

By interacting with receptors GPR35 and AHR, KYNA modulates interleukins re-lease, acting as anti-inflammatory agent. Moreover, the indoleamine 2, 3-dioxygenase 1, enzyme that catalyzes the formyl-kynurenine formation, is upregulated by a pro- inflammatory stimulus, and then KYNA concentration increases in order to control the inflammation [11,12,42].

Therefore, these antioxidant and anti-inflammatory properties of KYNA could reduce the endothelial vascular lesion caused by our rat aorta model of I/R, which explains how the molecule contributed to the recovery of the blood vessel functionality (both contraction and relaxation). Proteins related to the immune process and cell differentiation identified after KYNA treatment reinforce this mechanism of KYNA. Moreover, the protein profile expression was different between I/R and I/R+KYNA, with the treatment able to increase cellular processes, response to stimulus, and molecular function, categories that show the tissue repair.

Besides, the effect of KYNA in the acetylcholine-induced relaxation was more prominent compared to the one produced by sodium nitroprussiate. This data indicated the action of KYNA in endothelium-dependent mechanisms. One hypothesis is that the superoxide anions inactivate the endothelial nitric oxide (NO) and endothelium-derived relaxing factor, which reduced the aorta rings relaxation [43].

Similar to the results presented here, Laude et al. [44] observed that the ischemia followed by reperfusion decreased the coronary artery relaxation induced by acetylcholine, compared to animals without the procedure. The reperfusion, acute or chronic, was crucial for the injury extension and how severe was the vessel dysfunction.

For the contraction and relaxation, the neurotransmitters released from the vesicle’s mobilization are dependent on the intracellular calcium [45]. Proteins related to calcium metabolism were observed in the aorta treated with KYNA, as the Calcium-dependent secretion activator 1 (Q62717/Cadps), involved in exocytosis of vesicles filled with neurotransmitters and responsible for the regulation of catecholamines content inside dense vesicles [46].

In the same network, the receptor-type tyrosine-protein phosphatase (Q64605/ Ptprn2) was identified, a protein that acts in the accumulation of neurotransmitters, such as norepinephrine, in several organs, and plays a role in vesicle-mediated secretory processes [47]. These two proteins, expressed after KYNA treatment, can indicate regulation of the neurotransmitter release in both contraction and relaxation, as KYNA incubation restored the endothelial blood vessel function.

This interaction network also pointed out cadherin EGF LAG seven-pass G-type receptor 2 (Q9QYP2/Celsr2), highly expressed after KYNA treatment. This protein is important for cell/cell signaling during nervous system formation [48], which can contribute to the neuronal blood vessel recovery after the damage caused by the I/R. In this sense, neurotrophin-3 (P18280/Ntf3) was also identified, and it is related to survival of neurons. Yao et al. [49] discovered that this protein, when upregulated, increased growth factors (TGF-β1) in aortic valve interstitial cells. It is known to regulate fibroblast functions, such as cell survival, migration, and secretion of cytokines, besides smooth cell migration and MMP-9 production, important for the tissue recovery.

The expression of growth factors, such as nerve growth factor (NGF) and fibroblast growth factor-1 (FGF-1), was modulated by KYNA, essential for tissue repair and neuron’s recover [50,51].

It is important to mention that the STRING analysis constructed this network based on co-expressed proteins, which reinforces the hypothesis that KYNA is acting on the recovery of endothelium and neurons from the blood vessel, besides the regulation of neurotransmitters release.

Another protein-protein interaction was the one connected by pink edges, with proteins determined experimentally. One of them is the SH3 and multiple ankyrin repeat domains protein 3 (Shank3/Q9JLU4), involved in synapse formation, maturation, and maintenance of neurons, besides the interconnection of NMDA-type receptors and metabotropic glutamate receptors [52]. This protein has also been related to progressive vascular dilatation [53].

Connected with Shank3 there is Homer protein homolog 1 (Homer1/Q9Z214) that couples to the surface receptors to intracellular calcium release, which can be contributing to the neurotransmitter release, a process that needs calcium for vesicle exocytosis [54]. This protein was also found upregulated in coronary artery disease [55].

Also related to neurotransmitter release, there is the protein bassoon (Bsn/O88778) in this network, which is involved in the cytoskeletal matrix assembled at the pre-synapse site [56].

Another protein of this network is Ras/Rap GTPase-activating protein SynGAP (Syngap1/Q9QUH6) that regulates neuronal survival and regeneration, and it has been considered a potential therapeutic target after stroke due to this recovery effects on ischemia-induced neuronal injury [57].

All these proteins found after KYNA treatment in the aorta submitted to ischemia and reperfusion could explain a mechanism of tissue repair and control of neurotransmitter release, besides the scavenging reactive oxygen species, all those factors important for the recovery of the blood vessel function of contraction and relaxation. Therefore, this work shows an important role of KYNA in the post-ischemic condition, and the molecule can be considered in the treatment of I/R.

## 4. Materials and Methods

### 4.1. Reagents

All the employed reagents were purchased from Sigma (St. Louis, MO, USA) unless otherwise stated.

### 4.2. Animals

Sprague-Dawley rats (250–280 g) were obtained from Centro Multidisciplinar de Investigação Biológica (CEMIB), Universidade Estadual de Campinas (UNICAMP), and maintained in USF biotery under 24 °C, 12/12 h light dark cycle and food and water ad libitum. All the procedures were conducted according to the Universidade São Francisco Committee Ethics approval (# 001.02.2019).

### 4.3. Ischemia/Reperfusion Model

Rats were anesthetized by 4% isoflurane and the renal aorta was exposed and clamped for 30 min (ischemia induction) and then the blood flow was released for 5 min for reperfusion (I/R group, *n* = 6). In parallel, rats without the ischemia/reperfusion, but submitted to the surgery, were used as a comparison (sham group, *n* = 6).

After the procedure, the ischemic aorta was removed and dissecated and conducted to further studies, either functional or proteomics. Rats were then euthanized by a lethal concentration of the anesthetic.

### 4.4. Functional Studies

Abdominal aorta from I/R or sham group was cut into 3.0 cm. The rings were suspended in a 5 ml chamber connected to a recording system (Powerlab/400TM, AD Instruments, Dunedin, New Zealand) for measurement of isometric tension. The organ chamber was filled with Tyrode solution (composition in mM: NaCl 138, KCl 2.7, MgCl_2_ 1.0, CaCl_2_ 1.4, NaH_2_PO_4_ 0.36, NaHCO_3_ 12 and glucose 5.5), pH 7.4 at 37 °C, continuously bubbled with 95% O2 and 5% CO_2_. Each ring was placed under 5 mN tension and allowed to equilibrate for 20 min.

KYNA (100 µM) was incubated with the aorta rings (I/R + KYNA group; *n* = 6) for 30 min and then cumulative concentration response curves to phenylephrine (PHE; 1 nM to 100 μM), acetylcholine (ACh; 1 nM to 10 µM) or sodium nitroprusside (SNP; 1 nM to 10 µM) were obtained in the aorta, precontracted with PHE 10 μM. Data was analyzed by software Chart v7.0 (AD Instruments, Holliston, MA, USA). Each agonist was incubated with a ring from the same aorta, and the same animal.

Data analysis were performed by GraphPad Prism (GraphPad Software Inc., San Diego, CA, USA) to obtain concentration–response data fitted to a logistic function, pEC_50_ (the agonist concentration that produces a half-maximal response) and maximum effect of the agonist (E_max_).

### 4.5. Protein Identification

After being removed, aorta rings were placed into the organ chambers filled with Tyrode solution at 37 °C and aeration. KYNA (100 µM) was incubated for 30 min and then the vessels were immediately immersed in liquid nitrogen and macerated. The powder was solubilized in lysis buffer containing 8M urea and peptidase inhibitors (5 mM PMSF, 5 mM EDTA, 1 mM aprotinin). The solution was centrifuged at 10,000× *g* for 10 min at 4 °C and the protein concentration was determined in the supernatant by Bradford assay, following the manufactures’ instruction (BioRad, Hercules, CA, USA).

Samples of 50 µg of proteins were solubilized in 100 µL of 8 M urea and heated for 15 min at 80 °C. Then, it was added 100 mM dithiothreitol (DTT) and incubated for 30 min at 60 °C; after that, 300 mM iodoacetamide (IAA) was added and the material was incubated for 30 min at room temperature and protected from light. Tris-HCl (100 mM pH 8.5) was added to reach urea concentration to 2 M and 20 ng.µL^−1^ of trypsin (Proteomics Grade, from porcine pancreas, Sigma Aldrich) was incubated overnight at 37 °C. The enzymatic reaction was stopped adding by 5% formic acid (FA).

Ten microliter aliquots were inserted in a 50 mm × 1 mm C-18 column (Phenomenex, Torrance, CA, USA) coupled to UPLC system (Acquity UPLC M-class, Waters Co, Milford, MA, USA). The eluted content was automatically inserted in a Q-ToF Xevo GS mass spectrometer (Waters). Peptides were eluted in a linear gradient of 1–40% B (A = 0.1% FA; B: 90% acetonitrile in 0.1% FA), at 0.2 µL/min during 90 min. Spectra were acquired in positive ionization, in a range of 200 to 1800 *m/z* and FWHM 40000 resolution at 500 *m/z*. For the MS/MS analysis, argon collision energy was applied. The instrument control and data acquisition were conducted by MassLinx 4.2.

RAW files were directly loaded in the software Peaks Studio V7.0 (Bioinformatics Solutions Inc (BSI), Waterloo, ON, Canada) and the data was processed for protein identification, with the following parameters: MS and MS/MS error mass 10 ppm and 0.01 Da; methionine oxidation and carbamidomethylation as a variable and fixed modification, respectively; trypsin as the cleavage enzyme; maximum missed cleavages (3), maximum variable PTMs per peptide (3) and non-specific cleavage (both); the false discovery rate was adjusted to ≤0.5%; only proteins with the score ≥ 30 and containing at least 1 unique peptide were considered in this study. All data were analyzed against SwissProt Database, using Rat taxonomy.

### 4.6. Bioinformatic Analysis

The protein list was analyzed by the ID mapping from UniProt and results viewed in the Gene Ontology mode. The Protein-Protein Interaction Networks were constructed with STRING, by searching with the same list generated by the Peaks Software and *Rattus norvegicus* as organism.

### 4.7. Statistical Analysis

Data are shown as mean ± SEM. Statistical comparisons between groups and agonist concentrations were carried out using two-way ANOVA test for repeated measures, followed by Bonferroni post-test. A value of *p* < 0.05 was considered significant.

## 5. Conclusions

The KYNA incubation could recover the function of the rat aorta submitted to ischemia and reperfusion, with a mechanism of tissue repair and control of neurotransmitter release. These results show an important role of KYNA in the post-ischemic condition, and the molecule can be considered in the treatment of I/R.

## Data Availability

Data sharing not applicable.

## Supplementary Materials

Table S1: Proteins identified in rat aorta submitted to ischemia-reperfusion and in aorta with ischemia-reperfusion treated with 100 µM KYNA.

## Sample Availability

Samples of the compounds are not available from the authors.
